# Synapse-specific expression of mu opioid receptor long-term depression in the dorsomedial striatum

**DOI:** 10.1038/s41598-020-64203-0

**Published:** 2020-04-29

**Authors:** Braulio Muñoz, David L. Haggerty, Brady K Atwood

**Affiliations:** 1 Department of Pharmacology & Toxicology, Indiana University School of Medicine, Indianapolis, IN 46202 USA; 2 Stark Neurosciences Research Institute, Indiana University School of Medicine, Indianapolis, IN 46202 USA

**Keywords:** Neural circuits, Long-term depression

## Abstract

The dorsal striatum is a brain region involved in action control, with dorsomedial striatum (DMS) mediating goal-directed actions and dorsolateral striatum (DLS) mediating habitual actions. Presynaptic long-term synaptic depression (LTD) plasticity at glutamatergic inputs to dorsal striatum mediates many dorsal striatum-dependent behaviors and disruption of LTD influences action control. Our previous work identified mu opioid receptors (MORs) as mediators of synapse-specific forms of synaptic depression at a number of different DLS synapses. We demonstrated that anterior insular cortex inputs are the sole inputs that express alcohol-sensitive MOR-mediated LTD (mOP-LTD) in DLS. Here, we explore mOP-LTD in DMS using mouse brain slice electrophysiology. We found that contrary to DLS, DMS mOP-LTD is induced by activation of MORs at inputs from both anterior cingulate and medial prefrontal cortices as well as at basolateral amygdala inputs and striatal cholinergic interneuron synapses on to DMS medium spiny neurons, suggesting that MOR synaptic plasticity in DMS is less synapse-specific than in DLS. Furthermore, only mOP-LTD at cortical inputs was sensitive to alcohol’s deleterious effects. These results suggest that alcohol-induced neuroadaptations are differentially expressed in a synapse-specific manner and could be playing a role in alterations of goal-directed and habitual behaviors.

## Introduction

Alcohol exposure, both acute and chronic, leads to changes in brain function from the genetic level through to changes in cellular function and network alterations^1–4^. A common outcome of alcohol-induced cellular adaptations are changes in synaptic plasticity that likely underlie altered neurocircuit function^5,6^. One component of alcohol use disorder is a transition from flexible goal-directed alcohol use to more habitual or compulsive use and this is paralleled by a transition in the activity between the dorsomedial (DMS) and the dorsolateral striatum (DLS) subregions of the dorsal striatum^7,8^. The dorsal striatum is the input nucleus of the basal ganglia and is a key region involve in goal-directed learning and habit formation, where the DMS has a primary role in the control of outcome-driven behavior and learning and the DLS is important for stimulus-driven behavior and learning^7–12^. As alcohol use shifts from being outcome-driven to becoming more stimulus-driven and compulsive in nature during alcohol use disorder development, the balance of action control moves from DMS to DLS^7,8,13^. The specialized roles in action control of these two dorsal striatal subregions suggest that the neural circuitry of these two areas are differentially affected by ethanol during the development of alcohol use disorders^14–16^.

GABAergic medium spiny neurons (MSNs) are the striatal output neurons and comprise 95% of dorsal striatal neurons^17^. MSNs receive glutamatergic inputs from cortex, thalamus, basolateral amygdala, and cholinergic interneurons (CINs)^18–20^. Despite the similarities in neuronal composition and synapse types, the unique innervation of each striatal subregion^19^ indicates that these different patterns of input may be a critical component of determining the function of each region. It is therefore important to determine the “synaptic fingerprints” of these inputs: their origins, targeted cell types, protein composition, the types of plasticity they express, and how they are impacted by drugs of abuse. For example, it is increasingly understood that dorsal striatal synapses that express synaptic plasticity, specifically long-term synaptic depression (LTD) are critical for striatal-dependent behaviors and learning, but are also known to be altered by drugs of abuse, including alcohol^16,21–24^. Elucidating synapse-specific plasticity and the signaling mechanisms that underlie those differences, especially with a focus on differences between synapses within dorsal striatal subregions, will allow for a greater understanding of the mechanisms of drug-induced changes in behavior.

Our previous work has begun to characterize opioid receptor signaling at specific functional inputs to dorsal striatal MSNs^5,25^. The opioid system is broadly expressed in the dorsal striatum^26^ and is an important target for alcohol use disorder treatment^27^. In the dorsal striatum, MOR activation presynaptically reduces glutamate and GABA release and, through a polysynaptic mechanism, can also reduce dopamine release^5,25,28–31^. We previously found that the activation of MORs on cortical inputs produces a static (non-reversible) form of LTD (mOP-LTD) whereas MORs on thalamic inputs generate a labile (transient and reversible) type of plasticity that we operationally define as MOR-mediated short-term depression in both DLS and DMS^5,25,32^. Furthermore, we also found that mOP-LTD occurred at glutamatergic inputs to MSNs from local cholinergic interneurons (CINs) in DLS. However, when we used broad electrical stimulation to probe glutamatergic synapses, *in vivo* exposure to alcohol (ethanol) only disrupted mOP-LTD in the DLS and not the DMS^5^. Using optogenetic targeting mechanisms to probe specific synapses for MOR plasticity and sensitivity to ethanol, we found that the only type of cortical input that expresses mOP-LTD in DLS is the one originating in the anterior insular cortex (AIC)^5^, and this mOP-LTD was indeed sensitive to ethanol. In the DLS, mOP-LTD at CIN and MOR-mediated short-term depression at thalamostriatal synapses were unaffected by ethanol. Given the differential responses of mOP-LTD to ethanol between DLS and DMS, we hypothesized that mOP-LTD in the DMS occurs at different glutamatergic synapses than those in the DLS. We also predicted that probing of specific inputs might uncover a subpopulation of DMS glutamatergic synapses that would be affected by ethanol. In order to test these predictions, we used similar methodologies as our previous work, but with a focus primarily on the DMS^5^. Our primary finding was that mOP-LTD has a different profile in DMS than in DLS. It is expressed at multiple DMS inputs rather than one like in DLS. However, similar to DLS, inputs from cortex, but not other inputs, were sensitive to the plasticity-ablating effects of ethanol.

## Results

### AIC and OFC inputs do not express mOP-LTD in the DMS

**Figure 1:**
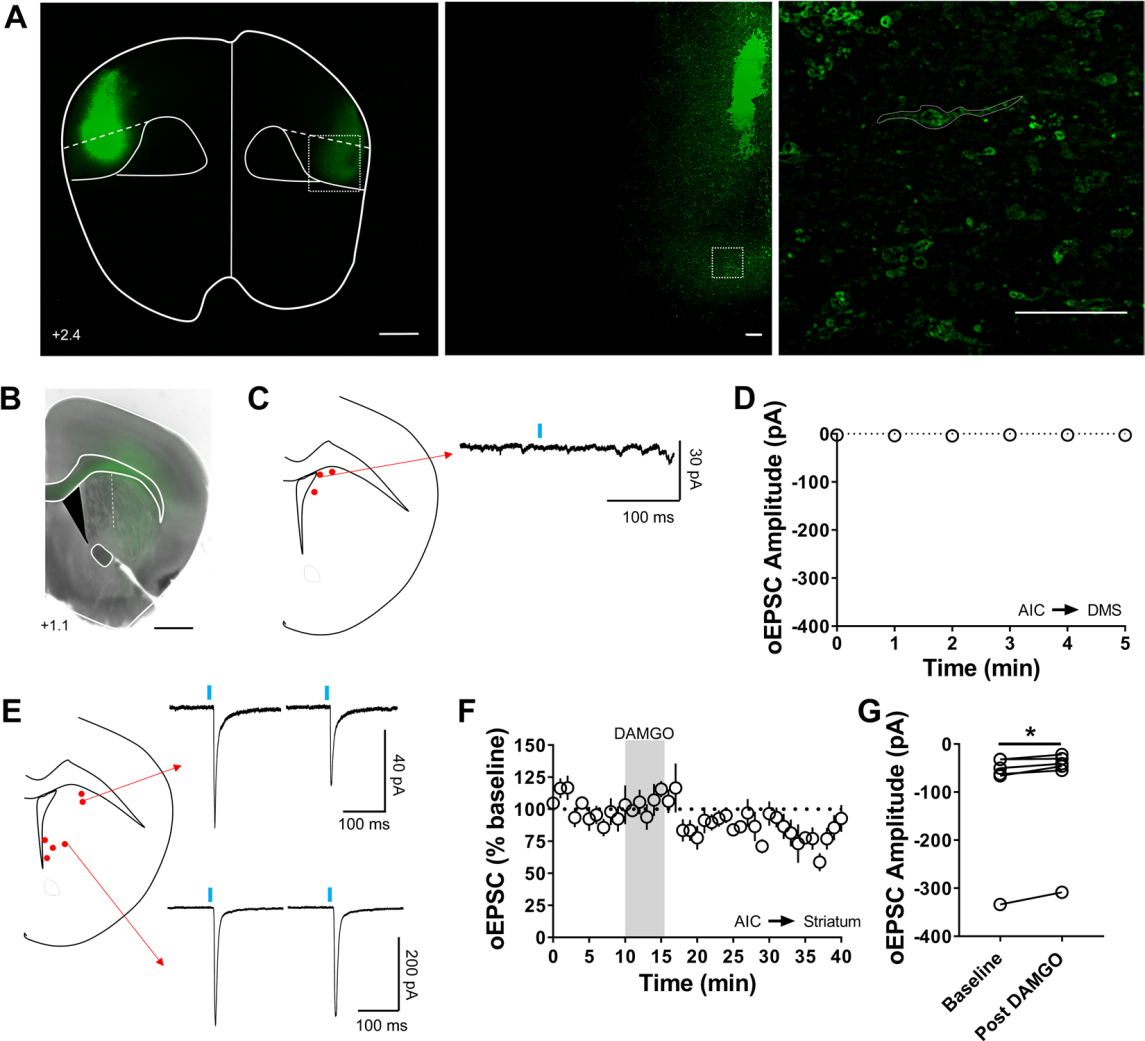
Anterior insular cortex does not send functional projections to the dorsomedial striatum. (**A**) Coronal brain slices from C57BL/6J mice showing the infection of cortical neurons in the AIC following infusion of an AAV9.hSyn.ChR2.eYFP vector (Scale bar from left to right = 1 mm, 100 µm and 25 µm). (**B)** Coronal brain slice from C57BL/6J mice showing the projection of AIC infected neurons to the dorsal striatum (Scale bar = 1 mm). (**C)** Schematic representation of 3 different sites of recordings, showing a representative trace. (**D)** 5 minute quantification of responses recorded from sites indicated in C demonstrating no generation of EPSCs evoked by optical stimulation (oEPSC). (**E–G)** Schematic representation of 2 lateral sites and 4 ventral sites of recordings, showing representative traces of oEPSCs from one lateral and one ventral site before (left traces) and after DAMGO treatment (0.3 μM, 5 min). The activation of MOR by DAMGO at these 6 sites reduced oEPSC amplitude and induced mOP-LTD. oEPSC amplitudes in MSNs within DMS were significantly reduced after DAMGO application (0–10 min baseline v. final 10 min of recording; paired t-test, P = 0.0191, t_5_ = 3.409, n = 6 neurons from 4 mice). Data represent mean ± SEM. *P < 0.05.

**Figure 2:**
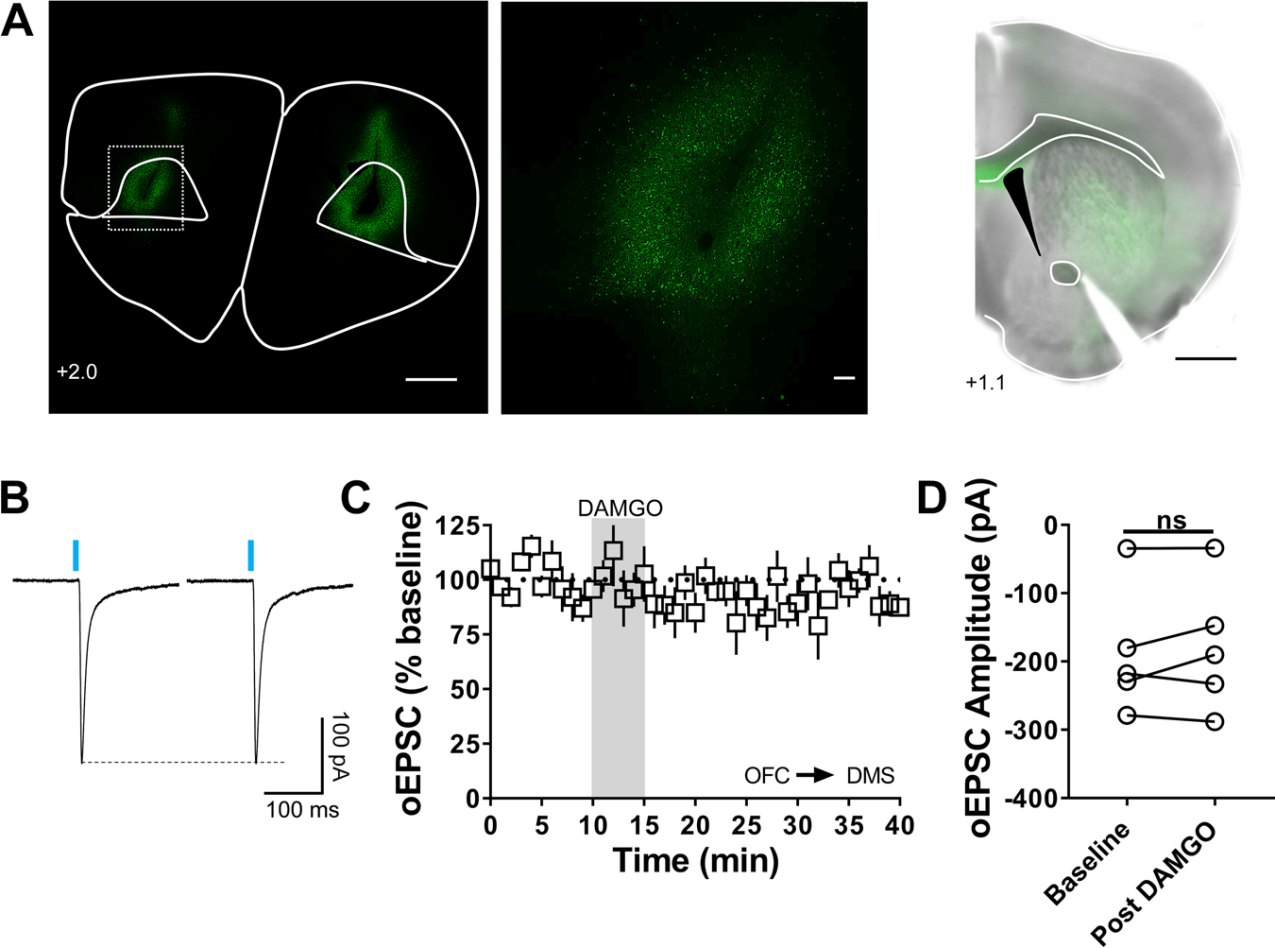
MOR activation does not produce LTD of excitatory transmission at orbitofrontal cortex inputs to the DMS (**A**) Coronal brain slice from C57BL/6J mice showing the infection of cortical neurons in the OFC following infusion of the AAV9.hSyn.ChR2.eYFP vector and the projection of those infected neurons to the dorsal striatum (Scale bar from left to right = 1 mm, 100 µm and 1 mm). (**B)** Representative optically evoked EPSC traces at baseline and after DAMGO (0.3 μM, 5 min) treatment. (**C,D**) DAMGO does not reduce oEPSC amplitude in MSNs of C57BL/6J mice at OFC inputs to DMS (0–10 min baseline v. final 10 min of recording; paired t-test, P = 0.6024, t_5_ = 0.5556; n = 6 neurons from 4 mice).

From our previous work, we broadly showed that cortical inputs express mOP-LTD in the DLS and DMS^5^. We previously probed specific synapses in the DLS to identify which synapses expressed mOP-LTD, but did not explore specific synapses in the DMS. We demonstrated that AIC inputs are the sole input that expresses mOP-LTD in the DLS^5^, however AIC has lower innervation of the DMS, compared with the DLS^19^. As a direct follow-up, we first explored mOP-LTD at AIC inputs to regions of the striatum more medial to our previous recordings^5^. To probe these AIC inputs, we expressed Channelrhodopsin2 (ChR2) in AIC neurons (Fig. 1A), made brain slices containing DMS and activated these AIC inputs using optical stimulation. We activated MORs by bath application of the agonist DAMGO (0.3 μM) for 5 minutes. AIC inputs did not produce any optically-evoked excitatory postsynaptic current (oEPSCs) in the most dorsomedial portion of the area where we typically perform DMS recordings (Fig. 1B–D). However, when we changed the recording site to slightly more lateral or ventral portions of the dorsal striatum, we were able to evoke oEPSC responses (Fig. 1E). Interestingly, those AIC inputs to those adjacent regions expressed mOP-LTD (84 ± 5%; baseline: −96 ± 48 pA vs post-DAMGO: 84 ± 45 pA; Fig. 1F,G). This is consistent with our findings in the DLS and also indicates that AIC does not send functional projections to the most dorsomedial part of the DMS. Our previous work also demonstrated that mOP-LTD and cannabinoid receptor-mediated LTD can functionally interact and that cannabinoid-LTD is expressed at OFC inputs to DMS^16,25^. Therefore, we predicted that MOR would also mediate LTD at OFC inputs. To our surprise, we found that OFC projections to the DMS did not express mOP-LTD (102 ± 7%; baseline: −190 ± 54 pA vs post-DAMGO: −186 ± 55 pA; Fig. 2B–D). These results indicated that, based on our previous work, two of the most likely sources of mOP-LTD in the DMS did not in fact express this form of plasticity leading us to explore additional sources.

### mOP-LTD is expressed at mPFC and ACC inputs to the DMS

**Figure 3:**
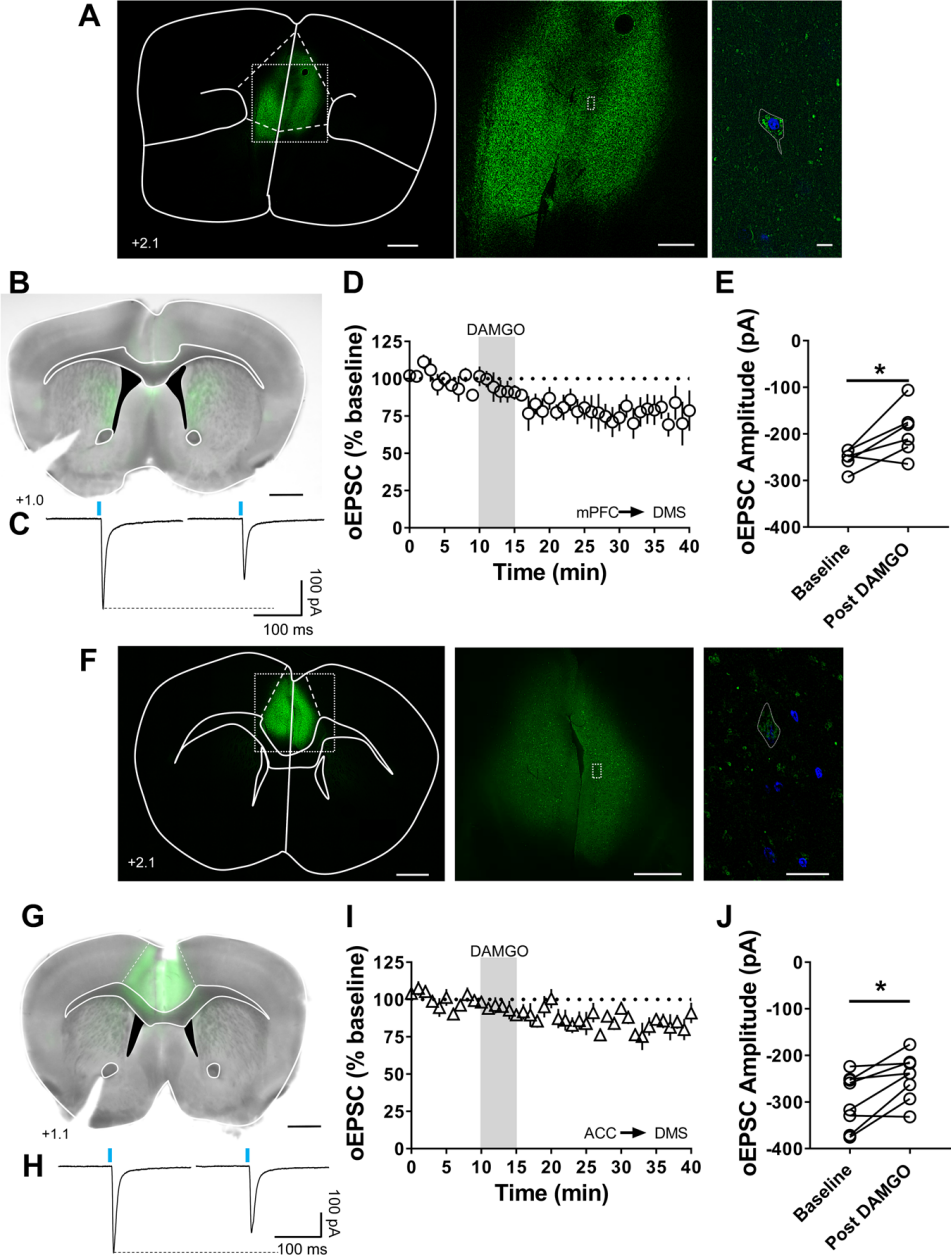
mOP-LTD occurs at medial prefrontal and anterior cingulate cortex inputs to the DMS. (**A,B**) Coronal brain slice showing the expression of the infused AAV9.hSyn.ChR2.eYFP in the mPFC and the projection of those infected neurons to the dorsal striatum (A: Scale bar from left to right = 1 mm, 500 µm and 10 µm; B: Scale bar: 1 mm). (**C)** Representative oEPSC traces in the DMS of C57BL/6J mice, before and after DAMGO application (0.3 μM, 5 min). (**D,E)** mOP-LTD at mPFC inputs occurs after the application of DAMGO. mOP-LTD is defined as the long-lasting reduction in oEPSC amplitudes after DAMGO (0–10 min baseline v. final 10 min of recording; paired t-test, P = 0.0313, t_5_ = 2.965, n = 6 neurons from 4 mice). (**F,G)** Coronal brain slice showing the expression of the infused AAV9.hSyn.ChR2.eYFP in the ACC and the projection of those infected neurons to the dorsal striatum (F: Scale bar from left to right = 1 mm, 500 µm and 10 µm; G: Scale bar: 1 mm). (**H)** Representative oEPSCs traces before and after DAMGO application. (**I,J)** mOP-LTD of ACC inputs occurs after the application of DAMGO (0.3 μM, 5 min). Post-DAMGO application reduced the oEPCS amplitudes (0–10 min baseline v. final 10 min of recording; paired t-test, P = 0.0104, t_7_ = 3.473, n = 8 neurons from 3 mice). Data represent mean ± SEM. *P < 0.05.

To determine if our previously identified corticostriatal mOP-LTD in DMS occurs at any other well-validated cortical inputs to the DMS, we tested mPFC and ACC inputs (Fig. 3A,B,F,G). Activation of MOR by DAMGO led to a decrease in oEPSC amplitude elicited by stimulation of inputs from mPFC (77 ± 8%; baseline: −253 ± 7 pA vs post-DAMGO: −194 ± 22 pA; Fig. 3C–E) and ACC regions (83 ± 5%; baseline: −298 ± 21 pA vs post-DAMGO: −247 ± 17 pA; Fig. 3H–J). Therefore, MOR activation can inhibit inputs to MSNs within DMS from at least two cortical areas and not just one as in the DLS^5^.

### MORs at BLA and CIN inputs induce mOP-LTD in the DMS

**Figure 4:**
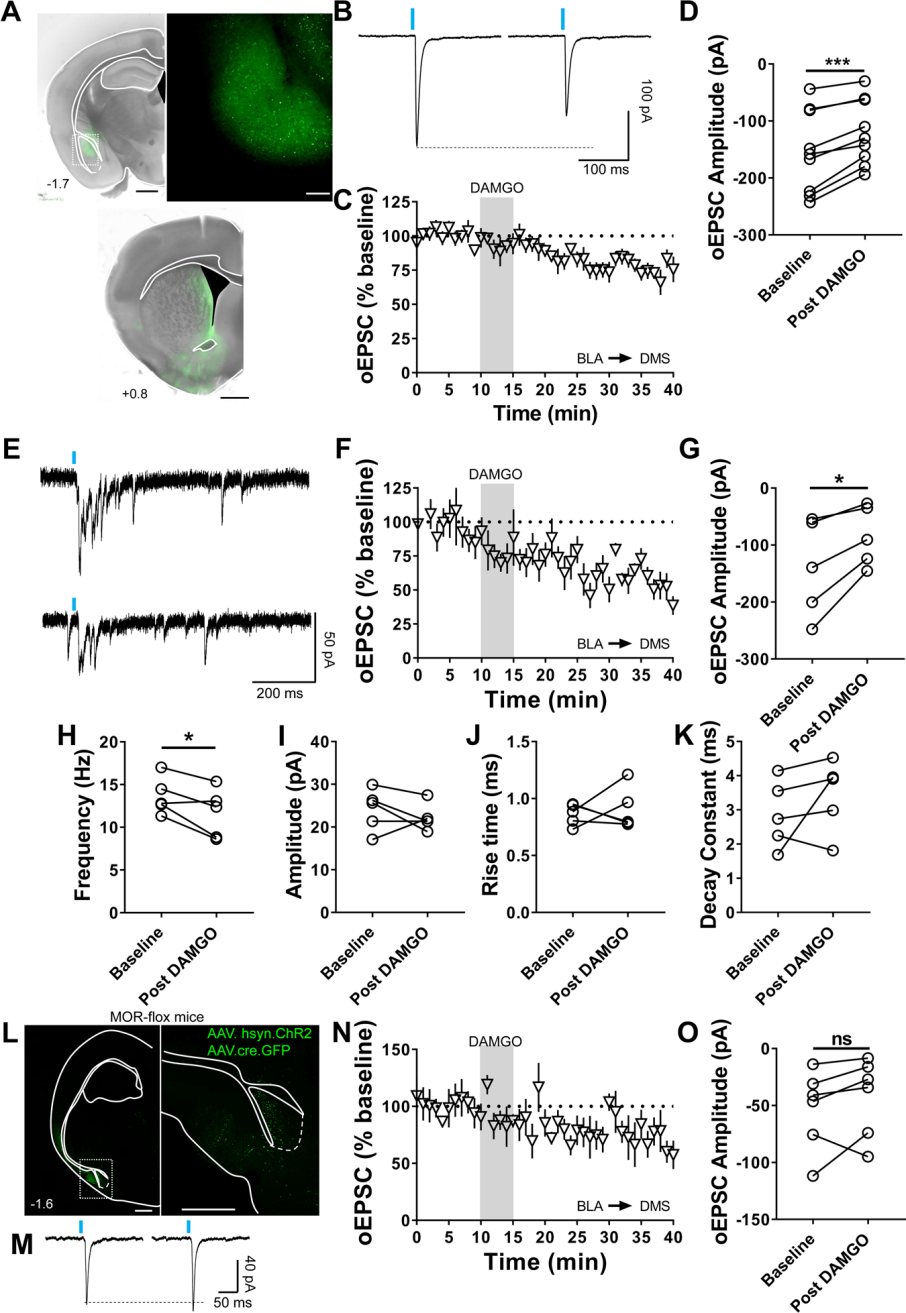
mOP-LTD occurs at basolateral amygdala inputs to the DMS. (**A**) Coronal brain slice showing the expression of the infused AAV9.hSyn.ChR2.eYFP in the BLA and the somas (Scale bar = 1 mm and 100 µm) and the projection of infected neurons to the dorsal striatum of C57BL/6J mice (Scale bar = 1 mm). (**B)** Representative oEPSCs traces at baseline and after DAMGO application (0.3 μM, 5 min). (**C,D)** DAMGO application induced mOP-LTD in the DMS as indicated by a significant reduction in oEPSC amplitude (0–10 min baseline v. final 10 min of recording; paired t-test, P = 0.0005, t_8_ = 5.648, n = 9 neurons from 5 mice). (**E)** Representative traces of Sr^2+^-mediated asynchronous oEPSCs from MSNs before and after DAMGO application. (**F,G)** Activation of MORs (DAMGO 0.3 μM, 5 min) at BLA inputs produces mOP-LTD in the DMS, even in the presence of Sr^2+^ (0–10 min baseline v. final 10 min of recording; paired t-test, P = 0.0202, t_4_ = 3.734, n = 5 from 2 mice). (**H–K)** Assessment of Sr^2+^-mediated asynchronous spontaneous EPSCs showed a significant reduction in the frequency of the sEPSCs after DAMGO application, but no effect on sEPSC amplitude, rise time or decay constant (0–10 min baseline v. final 10 min of recording; paired t-test, P = 0.0418, t_4_ = 2.953, n = 5 from 2 mice). (**L)** Coronal brain slice showing fluorescent protein expression in the BLA of a conditional MOR knockout (MOR-flox) mouse that was co-infused into BLA with AAV9.hSyn.ChR2.eYFP and AAV9.hSyn.cre.eGFP vectors (Scale bar = 1 mm and 500 µm). (**M)** Representative oEPSC traces at baseline and after DAMGO application. (**N,O)** In mice with MORs knocked out from BLA, DAMGO did not produce a significant change in oESPC amplitudes in DMS (0–10 min baseline v. final 10 min of recording; paired t-test, P = 0.2195, t_5_ = 1.403, n = 6 neurons from 2 mice). Data represent mean ± SEM. *P < 0.05, ***P < 0.001.

We were also interested in whether or not other glutamatergic synapses in DMS could express mOP-LTD. Our previous work indicated thalamic inputs do not express mOP-LTD in DMS^5^. BLA is another brain region that sends glutamatergic inputs to the DMS and is a known input that expresses alcohol-mediated plasticity^19,33^. To probe BLA inputs to DMS synapses, we expressed ChR2 in the BLA (Fig. 4A) and we found that DAMGO treatment was able to produce mOP-LTD at these inputs (77 ± 2%; baseline: −153 ± 24 pA vs post-DAMGO: −119 ± 19 pA; Fig. 4B–D). To explore if mOP-LTD is mediated by presynaptic MORs on BLA afferents, we replaced extracellular Ca^2+^ with Sr^2+^, and with this change we were able to induce asynchronous synaptic release of glutamate after BLA inputs were optically evoked in the DMS. As expected, measuring the evoked peak, we detected the presence of mOP-LTD from BLA after MOR activation, even in the presence of Sr^2+^ (59 ± 4%; baseline: −141 ± 38 pA vs post-DAMGO: −84 ± 24 pA; Fig. 4E–G). We next assessed the properties of the asynchronous events after the evoked peak, measures that can be used to ascertain whether drugs effects are presynaptic or postsynaptic^34^. We found a decrease in the frequency of the events after DAMGO application (baseline: 14 ± 1 Hz vs post-DAMGO: 12 ± 1 Hz; Fig. 4H) without changes in amplitude (baseline: 24 ± 2 pA vs post-DAMGO: 22 ± 1 pA; Fig. 4I), rise time (baseline: 0.9 ± 0.04 ms vs post-DAMGO: 0.9 ± 0.08 ms; Fig. 4J) or decay time (baseline: 3 ± 0.4 ms vs post-DAMGO: 3 ± 0.5 ms; Fig. 4K). To further characterize the role of presynaptic MORs from BLA, we used a conditional MOR knockout mouse (MOR-flox), co-injected with AAV-Cre and AAV-ChR2 vectors to knock out the expression of MORs specifically in the BLA and to allow for optogenetic stimulation of the BLA inputs to DMS (Fig. 4L). mOP-LTD appeared to be blunted at these BLA inputs (72 ± 11%, Fig. 4M,N) given that DAMGO failed to produce a significant decrease in oEPSC amplitude in these mice (baseline: −53 ± 14 pA vs post-DAMGO: −42 ± 14 pA; Fig. 4O). Altogether, we suggest that presynaptic MORs in the DMS are responsible for mOP-LTD expressed at BLA inputs.

**Figure 5:**
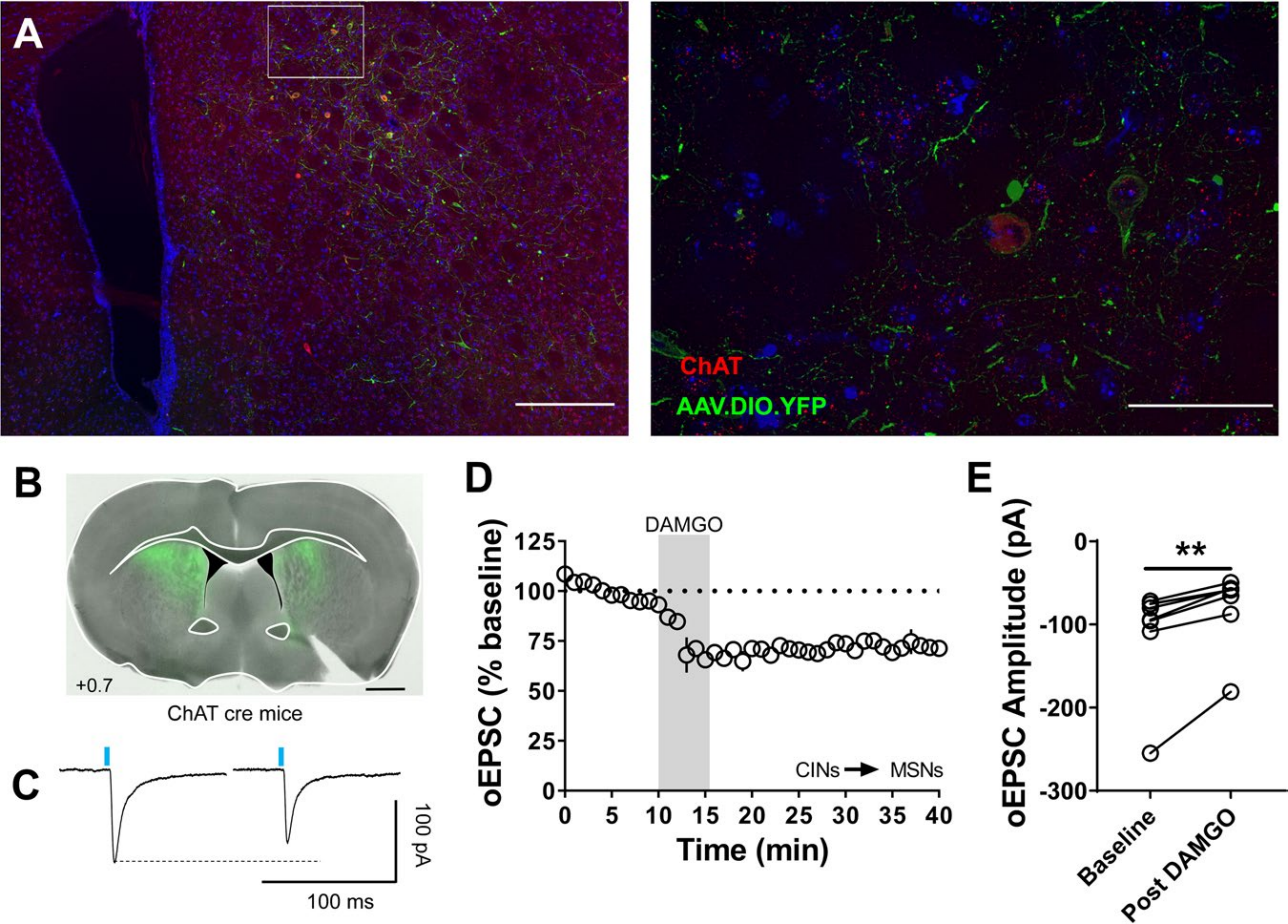
Cholinergic interneuron synapses on to DMS MSNs express mOP-LTD. (**A**) Coronal brain slice showing the infection of cholinergic interneurons (CINs) with the infused AAV.DIO.ChR2.eYFP (green) and the colocalization with anti-ChAT antibody (red) in the dorsal striatum of ChATCre mice (Scale bar = 250 µm and 50 µm). (**B)** Coronal brain slice showing the infection of cholinergic interneurons with the infused AAV.DIO.ChR2.eYFP vector in the dorsal striatum of ChATCre mice (Scale bar = 1 mm). (**C)** Representative traces of oEPSCs recorded in MSNS following optogenetic stimulation of CINs before and after DAMGO application. (D-E**)** Activation of MORs (DAMGO 0.3 μM, 5 min) at CIN inputs in ChATCre mice produces mOP-LTD in the DMS. DAMGO application reduced the oEPSC amplitudes (0–10 min baseline v. final 10 min of recording; paired t-test, P = 0.003, t_7_ = 4.429, n = 8 from 4 mice). Data represent mean ± SEM. **P < 0.01.

Given that, we previously found that mOP-LTD occurred at CIN inputs to DLS MSNs^5^, we also explored this possibility in the DMS as well. Using ChATCre mice, we infused a cre-recombinase-dependent AAV-ChR2 vector to express ChR2 only in CINs in the DMS. We found that the AAV.DIO.ChR2 vector injected in the DMS specifically infected CINs (Fig. 5A). Functionally, similar to DLS, we found that activation of CINs produces small oEPSCs in MSNs, but these synapses can still express mOP-LTD in the DMS (72 ± 2%; baseline: −107 ± 22 pA vs post-DAMGO: −77 ± 15 pA; Fig. 5B–E). Our previous work indicates that it is likely MORs expressed in CINs themselves that mediate this mOP-LTD^5^. Therefore, mOP-LTD is not only expressed at cortical inputs but also at BLA and CIN inputs on to MSNs in DMS.

### MORs on mPFC, ACC and BLA inputs are indispensable for mOP-LTD

**Figure 6:**
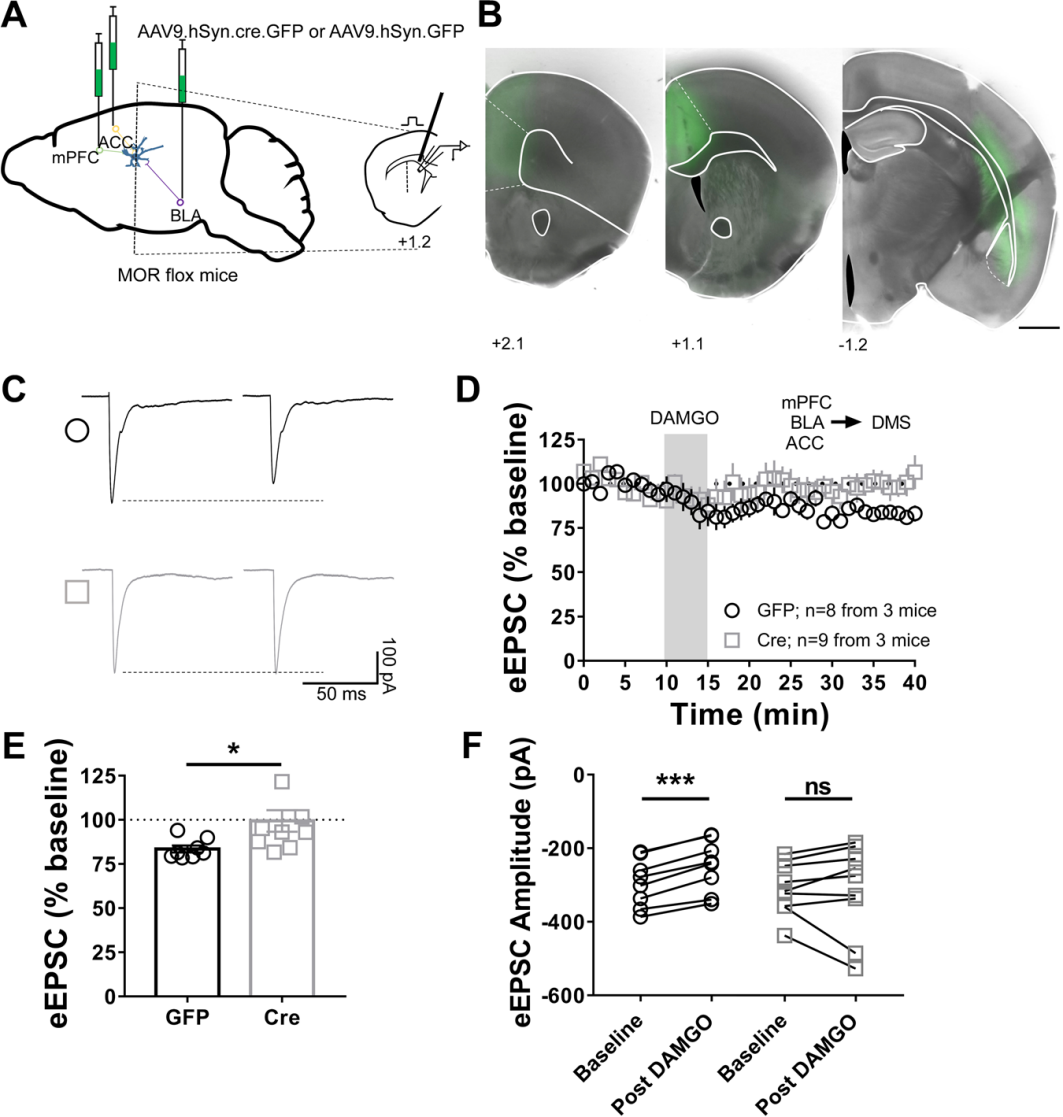
MORs on mPFC, ACC and BLA inputs are indispensable for the induction of mOP-LTD in the DMS. (**A**) Schematic figure of the triple injection paradigm enabling knock out of MOR expression in mPFC, ACC and BLA in MOR-flox mice. An AAV vector encoding for either cre-recombinase (AAV.hSyn.cre) or eGFP (AAV.hSyn.eGFP) was injected 8 weeks prior to recordings. (**B)** Coronal brain slice showing the infection of cortical and BLA neurons (Scale bar = 1 mm). (**C)** Representative electrically-evoked synaptic traces from AAV-eGFP-infused (open black circle) and AAV-Cre-infused (open grey square) mice, obtained in the DMS before and after DAMGO (0.3 μM, 5 min) showing the loss of mOP-LTD mediated by mPFC, ACC and BLA MORs. (**D,E)** After DAMGO application, eGFP-infected brain slices show mOP-LTD, but not brain slices from mice that were infused with AAV-Cre (last 10 minutes, Welch’s t-test, P = 0.0326). (**F)** eEPSC amplitudes were reduced after DAMGO application only in AAV-eGFP-infused mice, and not in AAV-Cre-infected MOR-flox mice, indicating that MORs from mPFC, ACC, and BLA are indispensable for MOR-mediated glutamatergic depression in the DMS (0–10 min baseline v. final 10 min of recording; paired t-test, eGFP: P < 0.0001, t_7_ = 11.24, n = 8 from 3 mice; cre: P = 0.8712, t_8_ = 0.1674, n = 9 from 3 mice). Data represent mean ± SEM. *P < 0.05, ***P < 0.001.

Our data to this point indicated that MOR activation was sufficient to induce mOP-LTD at corticostriatal and BLA-DMS inputs. To demonstrate that MORs on mPFC, ACC and BLA inputs are indispensable for mOP-LTD, we used MOR-flox mice, injected with AAV-Cre vectors to ablate the expression of MORs specifically in the mPFC, ACC and BLA (Fig. 6A,B). Here we used electrical stimulation for our recordings in the DMS (Fig. 6A), and found that DAMGO application did not produce mOP-LTD in AAV-Cre injected mice (99 ± 6%; Fig. 6C–F), as evidenced by no significative changes in eEPSC amplitudes (baseline: −309 ± 24 pA vs post-DAMGO: 313 ± 41 pA; Fig. 6F), but was present in AAV-eGFP injected control mice (83 ± 2%; Fig. 6C–F), as evidenced by a significant decrease in eEPSC amplitudes (baseline: −294 ± 23 vs post-DAMGO: 248 ± 25 pA; Fig. 6F). Therefore, presynaptic MORs on mPFC, ACC and BLA inputs are both indispensable and sufficient to induce mOP-LTD in the DMS.

### The effects of in vivo exposure to ethanol on mOP-LTD are specific to corticostriatal synapses

**Figure 7:**
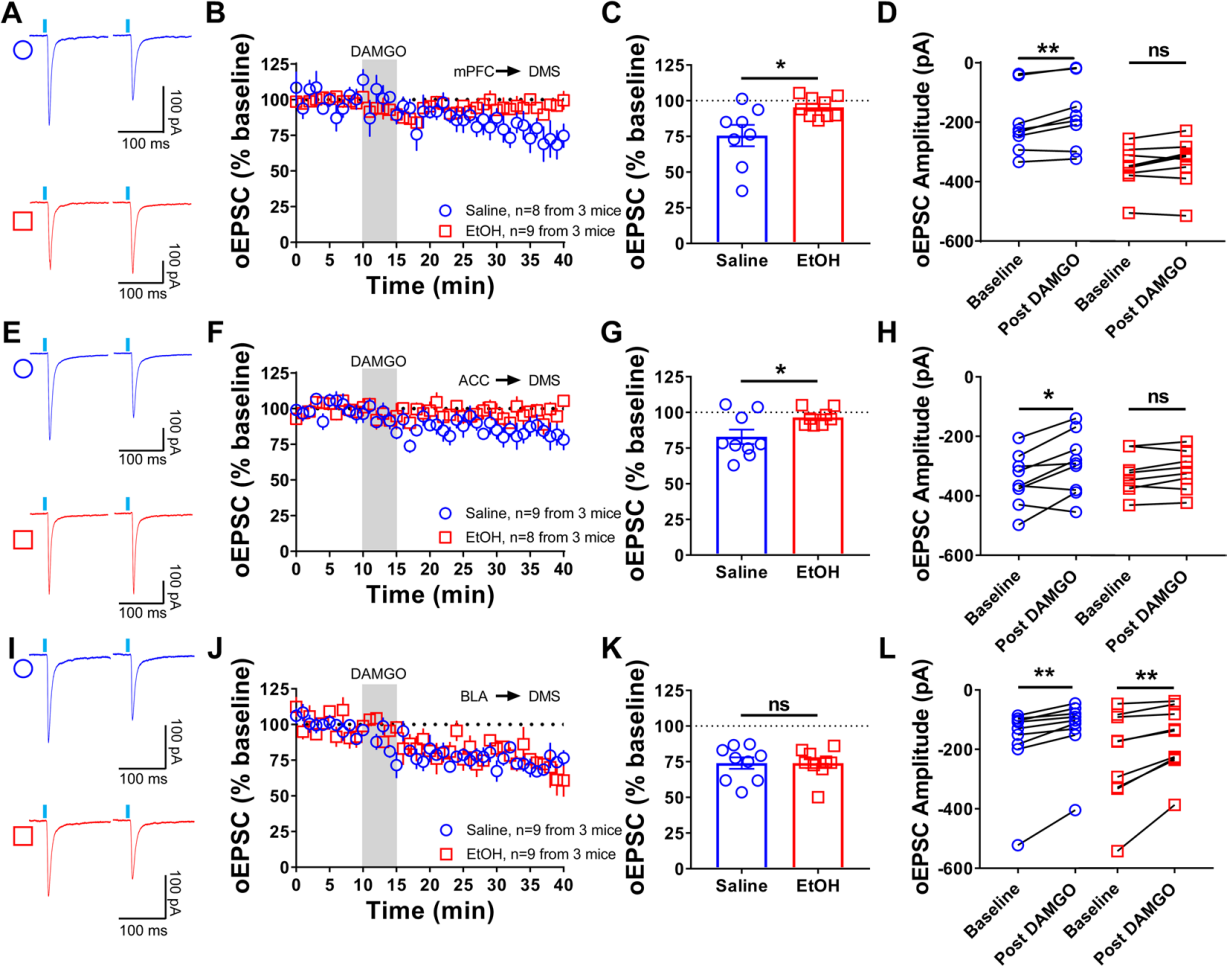
A single *in vivo* ethanol exposure prevents induction of mOP-LTD at mPFC and ACC inputs, but not at BLA synapses. C57BL/6J mice that were previously infused with AAV.hSyn.ChR2 in mPFC, ACC or BLA were injected (intraperitoneal) with saline or ethanol (EtOH, 2 g/kg). 24 h after this injection, oEPSCs in MSNs from the DMS were recorded. (**A)** Representative oEPSC traces from mPFC input stimulation in the DMS before and after DAMGO (0.3 μM, 5 min) application in saline- (blue traces) and ethanol- (red traces) injected mice. (**B,C)** Ethanol blunted mOP-LTD induced by DAMGO (0.3 μM, 5 min) from mPFC synapses in the DMS (last 10 minutes, Welch’s t-test, P = 0.0355). (**D)** Reduction of oEPSC amplitudes occurred only in saline-injected mice (baseline v. final 10 min of recording; paired t-test, P = 0.007, t_7_ = 3.768, n = 8 from 3 mice), but not in ethanol-injected ones (P = 0.068, t_8_ = 2.1, n = 9 from 3 mice). (**E)** Representative oEPSC traces following ACC input stimulation in the DMS before and after DAMGO application in saline- (blue traces) and ethanol- (red traces) injected mice. (**F,G)** Ethanol disrupted mOP-LTD induced by DAMGO (0.3 μM, 5 min) from ACC synapses in the DMS (Welch’s t-test, P = 0.0304). (**H)** Saline-injected mice expressed normal mOP-LTD in the DMS with a reduction in oEPSC amplitudes after DAMGO (0–10 min baseline v. final 10 min of recording; paired t-test, P = 0.0124, t_8_ = 3.211, n = 9 from 3 mice), but ethanol-injected mice showed disrupted mOP-LTD, with no difference in oEPSC amplitudes (P = 0.0939, t_7_ = 1.938, n = 8 from 3 mice). (**I)** Representative oEPSC traces following BLA input stimulation in the DMS before and after DAMGO (0.3 μM, 5 min) application in saline- (blue traces) and ethanol- (red traces) injected mice. (**J,K)** Ethanol does not affect mOP-LTD induced by DAMGO at BLA inputs (0.3 μM, 5 min) in the DMS (final 10 minutes, unpaired Student’s t-test, P = 0.9993, t_16_ = 0.0009). (**L**) Both treatments, saline (0–10 min baseline v. final 10 min of recording; paired t-test, P = 0.0036, t_8_ = 4.057, n = 9 from 3 mice) and ethanol (P = 0.0056, t_8_ = 3.759, n = 9 from 3 mice), do not affect mOP-LTD at BLA inputs. Data represent mean ± SEM. *P < 0.05, **P < 0.01.

We previously reported that mOP-LTD in MSNs within the DMS was not affected by *in vivo* ethanol exposure^5^, but we just explored those effects using broad electrical stimulation within DMS that would not be selective for any specific input^5^. Therefore, we tested the impact of *in vivo* ethanol exposure (2.0 g/kg; intraperitoneal, i.p.) on the expression of mOP-LTD at mPFC, ACC, and BLA inputs to the DMS. Mice injected with saline (i.p.) 24 h before harvesting tissue showed normal mOP-LTD at all these DMS synapses (mPFC: 76 ± 8%, Fig. 7A–D; ACC: 83 ± 5%, Fig. 7E–H; BLA: 74 ± 4%, Fig. 7I–L) following bath application of DAMGO. Interestingly, in mice injected with ethanol 24 h before harvesting tissue, mOP-LTD was ablated at the mPFC and ACC inputs (mPFC: 95 ± 2%; ACC: 97 ± 2%; Fig. 7A–H). However, ethanol pre-exposure did not influence mOP-LTD at the BLA-DMS inputs (74 ± 3%; Fig. 7I–L). We and others have demonstrated that *in vivo* ethanol exposure does not alter basal synaptic transmission^5,35^, but to rule out postsynaptic effects of ethanol, we also measured MSN intrinsic excitability. Using current clamp recordings, we found no differences between saline and ethanol injected mice in the frequency of the action potentials (Supplementary Fig. S1A,B), and in the others membrane parameters evaluated (Supplementary Fig. S1C–G). In sum, the data indicate that a single *in vivo* ethanol exposure is able to disrupt the induction of mOP-LTD at corticostriatal synapses, but not at BLA inputs to DMS.

## Discussion

Our previous work^5^ demonstrated the synapse-specificity of mOP-LTD at AIC synapses on to DLS MSNs and its singular disruption by ethanol. Using the strictest anatomical definition of what can be classified as “DMS,” we did not find evidence of functional AIC inputs to this striatal subregion. This aligns with anatomical analyses performed by others^19,36^. Interestingly we found that mOP-LTD does occur at AIC inputs to regions of dorsal striatum that are adjacent to the DMS, closer to ventral striatum (nucleus accumbens) and DLS. In contrast to DLS with its lone mOP-LTD-expressing input, our study demonstrated that mOP-LTD occured at multiple inputs to DMS. MOR activation on specific corticostriatal (ACC and mPFC) and BLA glutamatergic inputs to the DMS all express mOP-LTD. Consistent with our previous work though, only the corticostriatal synapses were sensitive to *in vivo* ethanol exposure. While we cannot completely rule out mOP-LTD at other minor inputs to DMS, we conclude that the major source of mOP-LTD in the DMS is at inputs from mPFC, ACC and BLA, since we demonstrated that the ablating of MOR expression from those areas is sufficient to disrupt mOP-LTD expression in the DMS. The current report together with our previous data demonstrate that MOR-mediated corticostriatal LTD is completely presynaptic using a variety of measures, including paired pulse ratio assessments and measures of spontaneous EPSCs/miniature EPSCs^5,32^. In our previous work and here, we have not found data to suggest that there are target cell type-specific forms of mOP-LTD plasticity, but we acknowledge that we have not specifically tested this possibility here, and this is something that will require further assessment in the future.

We previously demonstrated that cannabinoid receptor-mediated LTD occurs at OFC inputs to DMS^16^, but in the present study we found that OFC-DMS synapses did not respond to MOR agonist treatment. These data, in combination with our findings from DLS^5^, suggest that OFC inputs lack expression of presynaptic MORs. This was surprising to us as we had previously found that mOP-LTD and cannabinoid-LTD are mutually occlusive with one another^25^ and thought it likely that since OFC inputs to dorsal striatum express cannabinoid-LTD, that we would also find mOP-LTD at these synapses^16^. Future work will need to resolve how mOP-LTD and cannabinoid-LTD interact, if they are indeed expressed at the same synapses or if they display a heterosynaptic relationship.

In addition to identifying mOP-LTD at specific DMS inputs, we also identified the presence of mOP-LTD at CIN inputs on to MSNs within the DMS, consistent with our previous work^5^. CINs are an important component of the network balance of the striatum^37^, and can affect corticostriatal cannabinoid-LTD^38^, thalamic glutamate release^39^, and dopamine tone^40^. In addition to our work, others have reported on the role of MOR in modulating CIN activity^30,41^. Future work will need to explore the specific role of CIN-driven glutamate release and the role of MOR signaling in these neurons.

While many others have reported the effects of MORs on glutamatergic synaptic transmission in dorsal striatum^5,25,28,29,31^, until recently, only one other study has specifically explored regulation of DMS transmission via presynaptic MORs^28^. These investigators found that the only source of MOR-mediated depression of glutamatergic transmission in the DMS occurs at thalamic inputs^28^. Our findings contradict this study, but there are a few possible explanations for the different findings. One possible explanation is the duration of DAMGO exposure. The other study did not report the duration of agonist exposure and representative current traces implied that DAMGO might be having some effect that was also not reversed by the MOR antagonist CTAP. However no time course was provided so it was not possible to evaluate whether MORs were sufficiently engaged to induce LTD. Another likely explanation is methodological. Our recording conditions only block GABA_A_ receptor-mediated transmission, whereas this other study used a cocktail of metabotropic GABA, glutamate, and acetylcholine receptor antagonists in addition to antagonists of GABA and nicotinic acetylcholine ionotropic receptor antagonists. Our study here does not rule out the necessity of other neurotransmitters being co-factors for mOP-LTD and indeed our earlier work suggested that mGluR5 transmission might be an important element of opioid-LTD in DLS, at least for the form mediated by endogenous opioids^25^. Much more work is required to decipher the mechanisms of mOP-LTD including if induction of opioid-LTD by endogenous opioids in DMS utilizes similar mechanisms as in DLS^25^.

We previously reported that mOP-LTD in the DMS was not affected by ethanol exposure^5^. The critical difference here is that the previous study did not explore synapse-specific MOR plasticity like we performed here, but rather broadly probed glutamate transmission. Using local electrical stimulation, it is difficult to ascertain the relative contribution of various cortical, thalamic, amygdalar, and cholinergic interneuron inputs to any given MSN. Our previous data and the data presented here show that ethanol has synapse-type-specific effects: ethanol disrupted mOP-LTD at corticostriatal, but not other synapse types. Altogether, these results could explain the lack of ethanol effects on mOP-LTD in the DMS in our previous work, possibly because the ethanol-insensitive MOR-mediated plasticity at non-cortical inputs to DMS MSNs blunted the observation of ethanol’s effects on mOP-LTD at corticostriatal synapses, although additional work will need to be performed to validate this hypothesis. Taking these data together with our previous work indicates that there is some mechanism that makes MOR plasticity at corticostriatal synapses sensitive to disruption by ethanol, but MOR plasticity at thalamic, amygdalar, and CIN glutamatergic inputs insensitive. Future work will determine if ethanol needs a particular synaptic environment to affect mOP-LTD and if this is due to different mechanisms of MOR plasticity at these different synapses or if it is due to upstream effects of ethanol on cortex. Others have demonstrated that ethanol is able to alter LTD mediated by other presynaptic G_i/o_-coupled GPCRs such as cannabinoid-LTD in DLS^15,24,42^ and mGluR2-LTD in DLS and DMS of adolescent mice^43^. Ethanol is also known to disrupt other forms of LTD at multiple other synapse types in the brain^6^. It will be of great interest if ethanol has a common mechanism of action at all of these different synapses.

Our data provide some interesting possibilities for how ethanol may influence DMS-mediated goal-directed behavior given that its effects, in relation to mOP-LTD, are limited to cortical afferents. A loss of LTD at specific cortical inputs could permit larger synaptic drive from these cortical regions resulting in greater control over striatal activity. For example, one study showed that LTD induction at mPFC synapses within the DMS decreased alcohol-seeking behavior, while LTP induction increased this behavior^22^. A loss of mOP-LTD at these same synapses may be akin to the LTP conditions and may therefore play a role in increased alcohol-seeking behavior in ethanol-dependent animals. Even though mOP-LTD at BLA inputs was insensitive to ethanol exposure, BLA inputs may have a role in ethanol and drug relapse and therefore MORs may modulate these amygdalostriatal-mediated behaviors^44,45^. In the future, it will also be important to not only investigate the origins of MOR-sensitive synapses, but also the afferent targets: D1- or D2-expressing MSNs (direct or indirect pathway respectively) given their different roles in DMS-mediated ethanol-related behaviors^46^.

In conclusion, in combination with our previous work, we have demonstrated that MORs appear to only regulate specific dorsal striatum synapses with each of these different regions expressing specific forms of MOR-mediated plasticity (LTD or short-term depression). In addition, some mOP-LTD-expressing synapses are sensitive to ethanol and others insensitive. Altogether these findings indicate that specific striatal glutamatergic synapses express unique complements of signaling processes that result in different types of plasticity being expressed that are differentially sensitive to drugs of abuse. The specific molecular machinery that creates these differences may be a “synaptic fingerprint” of drug-sensitive synapses. In the future, it will be important to further elucidate the synaptic fingerprints of not only MOR signaling at specific striatal synapses, but also other forms of plasticity, to mechanistically understand ethanol’s network effects in order to identify novel treatments for alcohol use disorder and drug addiction.

## Methods

All experiments were performed similar to our previous studies with some experiment-specific modifications^5,25,47,48^. These methods are described in brief below.

### Animals and materials

Animal care and experimental protocols for this study were approved by the Institutional Animal Care and Use Committee at the Indiana University School of Medicine and all guidelines for ethical protocols and care of experimental animals established by the NIH (National Institutes of Health, Maryland, USA) were followed. Male C57BL/6J mice were ordered from the Jackson Laboratory (Bar Harbor, Maine, USA). ChATCre transgenic mice were bred and genotyped in-house (Original stock strain: ChATCre: JAX #006410). Conditional MOR knockout mice (MOR-flox) were a generous gift from Dr. Jennifer Whistler (UC Davis) and have been previously described^5,49^. All transgenic mice used in these studies have been backcrossed to C57BL/6J mice for a minimum of 7 generations. The mice used in these studies were between PND 60–100 at the time of experimentation (with the exception of ChATCre mice that were ~PND 60–120 and MOR-flox AAV-cre-injected mice ~PND 105–126). Animals were group-housed in a standard 12-h light/dark cycle (lights on at 0800) at 50% humidity. Food and water were available *ad libitum*.

### Brain slice preparation

Mice were euthanized via decapitation under deep isoflurane anesthesia, and the brain was quickly excised and placed in an ice-cold cutting solution containing (in mM): 194 sucrose, 30 NaCl, 4.5 KCl, 1 MgCl_2_, 26 NaHCO_3_, 1.2 NaH_2_PO_4_, 10 Glucose saturated with a mixture of 95% O_2_ and 5% CO_2_, and sliced to a thickness of 280 μm on a vibratome (Leica VT1200S, Germany). Slices were transferred to an artificial cerebrospinal fluid (aCSF) solution containing (in mM): 124 NaCl, 4.5 KCl, 1 MgCl_2_, 26 NaHCO_3_, 1.2 NaH_2_PO_4_, 10 Glucose, 2 CaCl_2_ (310–320 mOsm) saturated with 95% O_2_/5% CO_2_ at 30 °C for 1 hr before being moved to room temperature. When ready for recording, slices were transferred to a recording chamber continuously perfused with aCSF solution saturated with 95% O_2_/5% CO_2_.

### Electrophysiology recordings

Whole-cell recordings of excitatory postsynaptic currents (EPSCs) in MSNs were carried out at 29–32 °C and aCSF was continuously perfused at a rate of 1–2 ml/min. Recordings were performed in the voltage clamp configuration using a Multiclamp 700B amplifier and a Digidata 1550B (Axon Instruments, Union City, CA). Slices were visualized on an Olympus BX51WI microscope (Olympus Corporation of America). MSNs were identified by their size, membrane resistance, and capacitance. Picrotoxin (50 μM) was added to the aCSF for recordings to isolate excitatory transmission. Patch pipettes were prepared from filament-containing borosilicate micropipettes (World Precision Instruments) using a P-1000 micropipette puller (Sutter Instruments, Novato, CA), having a 2.0–3.5 MΩ resistance. The internal solution contained (in mM): 120 CsMeSO_3_, 5 NaCl, 10 TEA-Cl, 10 HEPES, 5 lidocaine bromide, 1.1 EGTA, 0.3 Na-GTP and 4 Mg-ATP (pH 7.2 and 290–310 mOsm). MSNs were voltage clamped at −60 mV for the duration of the recordings. To detect asynchronous glutamatergic transmission, aCSF Ca^2+^ was replaced with Sr^2+^ (2 mM). For electrically evoked recordings, a twisted tungsten bipolar stimulating electrode (PlasticsONE, Roanoke, VA) was placed at the border of the overlying corpus callosum. eEPSCs were generated by a DS3 Isolated Current Stimulator (Digitimer, Ft. Lauderdale, FL) every 20 s and stimulus intensity was adjusted to produce stable electrically-evoked EPSCs (eEPSCs) of 200–600 pA in amplitude prior to the initiation of experimental recording. Data were acquired using Clampex 10.3 (Molecular Devices, Sunnyvale, CA). Series resistance was monitored and only cells with a stable series resistance (less than 25 MΩ and that did not change more than 15% during recording) were included for data analysis. Recordings were made 2–7 h after euthanasia.

### Viral injections

Male ChATCre mice were anesthetized with isoflurane and stereotaxically injected with the adeno-associated viral (AAV) vector, AAV9.EF1a.DIO.ChR2(H134R)-eYFP (Penn Vector Core/Addgene) to drive expression of the photosensitive cation channel, channelrhodopsin2 (ChR2), solely in CINs. Bilateral injections were made into dorsal striatum at coordinates A/P: +0.7, M/L: ±1.5, D/V: −3.1 (100 nl/injection, 25 nl/min infusion rate). Mice were allowed to recover for at least 2 weeks before brain slices were made for electrophysiological recordings. Male C57BL/6J mice were anesthetized with isoflurane and stereotaxically injected with the adeno-associated viral (AAV) vector, AAV9.hSyn.ChR2(H134R)-eYFP (Penn Vector Core/Addgene) to drive ChR2 expression in mPFC, OFC, ACC, BLA and AIC neurons. Bilateral injections were made into mPFC at coordinates A/P: +1.9, M/L: ±0.3, D/V: −2.3 (100 nl/injection, 25 nl/min infusion rate); OFC: A/P: +2.7, M/L: ±1.75, D/V: −2.25 (100 nl/injection, 25 nl/min infusion rate); ACC: A/P: +1.4, M/L: ±0.3, D/V: −1.75 (100 nl/injection, 25 nl/min infusion rate); BLA: A/P: −1.6, M/L: ±3.35, D/V: −4.5 (100 nl/injection, 12.5 nl/min infusion rate) and AIC: A/P: +2.4, M/L: ±2.3, D/V: −2.25 (50 nl/injection, 12.5 nl/min infusion rate).

To induce specific oEPSCs from BLA projection neuron MOR knockout mice, MOR-flox mice were anesthetized with isoflurane and stereotaxically injected with the combination of AAV9.hSyn.Cre.eGFP plus AAV9.hSyn.ChR2.eYFP (Penn Vector Core/Addgene). Bilateral injections were made into BLA at coordinates: A/P: −1.6, M/L: ±3.35, D/V: −4.5 (100 nl/injection, 12.5 nl/min infusion rate). MOR-flox mice were allowed to recover for at least 8 weeks to allow for adequate ablation of MOR expression before brain slices were made for electrophysiological recordings.

To produce triple mPFC, ACC and BLA projection neuron MOR knockout mice, MOR-flox mice were anesthetized with isoflurane and stereotaxically injected with AAV9.hSyn.Cre.eGFP or AAV9.hSyn.eGFP as control (Penn Vector Core/Addgene). Bilateral injections were made into mPFC, ACC and BLA at coordinates: mPFC: A/P: +1.9, M/L: ±0.3, D/V: −2.3 (100 nl/injection, 25 nl/min infusion rate); ACC: A/P: +1.4, M/L: ±0.3, D/V: −1.75 (100 nl/injection, 25 nl/min infusion rate) and BLA: A/P: −1.6, M/L: ±3.35, D/V: −4.5 (100 nl/injection, 12.5 nl/min infusion rate). MOR-flox mice were allowed to recover for at least 8 weeks to allow for adequate ablation of MOR expression before brain slices were made for electrophysiological recordings.

### Immunohistochemistry

ChATCre mice previously infused with AAV9.EF1a.DIO.ChR2(H134R)-eYFP vector (Penn Vector Core/Addgene) were overdosed with a ketamine/xylazine (87.5 mg/kg ketamine and 12.5 mg/kg xylazine) cocktail (0.1 ml/20 g mouse weight) and then perfused transcardially with cold (4 °C) phosphate-buffered saline (PBS) followed by 4% paraformaldehyde (PFA) in PBS. Brains were extracted and placed in cold 4% PFA in PBS for 24 hours and then in cold 30% sucrose in double-distilled water for an additional 24 hours. Using a vibratome, 50 µm coronal sections were collected in serial order in cold PBS. Free-floating slices were incubated in 5% normal goat serum (NGS) in phosphate-buffered saline + Triton X-100 (PBST) on an orbital shaker for 1 hour at room temperature (RT). Immunostaining for the Choline Acetyltransferase (ChAT) protein was accomplished by using a primary antibody directed against ChaT (Anti-Choline Acetyltransferase, Sigma, cat.no. AB144P, diluted 1:100) in 5% NGS in PBST overnight on an orbital shaker at 4 °C. Sections were washed in PBST (3 times for 10 minutes) and then incubated in a secondary antibody (Donkey anti-Goat IgG (H + L), Alexa Fluor 568, Life Technologies, cat. no. A-11057, diluted 1:200) in 5% NGS in PBST for 2 hours on an orbital shaker at RT. Sections were washed in PBST (3 times for 10 minutes) and then mounted onto slides. Slides were cover slipped using Vectashield Hard Mount Set Medium + DAPI (Vector Laboratories), dried overnight, and then sealed with clear nail polish. Immunofluorescent images were acquired using a microscope (Keyence BX-800) with a 20x and 60x objective.

### Optogenetic recordings

AAV-DIO-ChR2-injection in ChATCre mice allows for targeted recombination manipulations only within CINs^5,50,51^. They were used in the present study to express ChR2 in dorsal striatal CINs. AAV-ChR2 injection in C57BL/6J mice was performed to target ChR2 expression to inputs from mPFC, OFC, ACC, BLA and AIC to DMS. AAV-ChR2 injection in MOR-flox mice was performed to target ChR2 expression to inputs from BLA to DMS. Optically-evoked EPSCs (oEPSCs) in MSNs were produced in brain slices using 470-nm blue light (5-ms exposure time) delivered via field illumination through the microscope objective. Light intensity was adjusted to produce stable oEPSCs of 200–600-pA amplitude prior to experimental recording. oEPSCs were evoked every 30 s. Prior to recording, brain slices were imaged via an Olympus MVX10 microscope (Olympus Corporation of America) to verify eYFP-tagged ChR2 expression in injected ChATCre+, MOR-flox and C57BL/6J mice or properly localized cre-recombinase expression (indicated by eYFP fluorescence). Brightfield images were used to draw boundaries of anatomical landmarks in low-magnification images. High-magnification representative images were acquired using a microscope (Keyence BX-800) with 4x and 20x objectives. Drawn outlines of anatomical boundaries in high-magnification representative images were determined by comparing the images’ maximum intensities to a mouse brain atlas (Franklin and Paxinos, The Mouse Brain, Elsevier, 2007). Representative images of brain slices in figures also indicate A/P coordinates from Bregma.

### Ethanol exposure

Mice (PND 80–100) received intraperitoneal (i.p.) injections of 0.9% NaCl saline (10 ml/kg) or 2 g/kg ethanol. Brain slices were obtained for electrophysiological recordings (as described above) 24 hr after ethanol injection. Animals were randomized to treatments, but the experimenter was not blinded to treatments.

### Reagents

We used the MOR agonist [D-Ala^2^, NMe-Phe^4^, Gly-ol^5^]-enkephalin (DAMGO; H-2535, Bachem), ethanol (ethanol, E7148, Sigma-Aldrich) and GABA_A_ receptor antagonist picrotoxin (PTX, P1675, Sigma-Aldrich). Other reagents used for making solutions were purchased from Sigma-Aldrich or Fisher Scientific.

### Sample size

The target number of samples in each group for behavioral, biological, and electrophysiological experiments was determined based on numbers reported in published studies^5,25^.

### Replication

All sample sizes indicated in figures for electrophysiological experiments represent biological replicates. N equals the number of slices recorded. 1 neuron was recorded from per brain slice.

### Data analyses

Unless otherwise indicated, data are presented as the mean ± SEM. The analyses of normally distributed data were performed using two-tailed unpaired or two-tailed paired Student’s t tests following an F test to confirm similar variances. Non-normally distributed data were analyzed using two-tailed Welch’s t test for unpaired data (Figs. 6E, 7C,G). Data that were analyzed using this test are indicated in the figure legends. Statistical analyses were performed with Prism 7 (GraphPad, La Jolla, CA). The level of significance was set at *P* < 0.05 for all analyses. Representative traces are the average baseline EPSC (1–10 min) and average post-treatment EPSC of final 10 min of recording. Exclusion of individual data points was determined using a ROUT outlier calculator (Q = 1%) included in the Prism 7 software package.

## Supplementary information

Supplemental Materials.

## Data Availability

All data are available from the authors upon reasonable request.
