# Supplementary material for: Synapse-specific expression of mu opioid receptor long-term depression in the dorsomedial striatum
Source: Sci Rep. 2020 Apr 29;10:7234. doi: 10.1038/s41598-020-64203-0 (PMC7190836; doi:10.1038/s41598-020-64203-0)
Supplement: Supplementary file 1 — Supplemental Materials. [file 41598_2020_64203_MOESM1_ESM.pdf]

Synapse-specific expression of mu opioid receptor long-term depression in the dorsomedial striatum

Braulio Muñoz[1], David L Haggerty[1] and Brady K Atwood[1,2]

[1]Department of Pharmacology & Toxicology, [2]Stark Neurosciences Research Institute, Indiana University School of Medicine, IN, USA 46202.

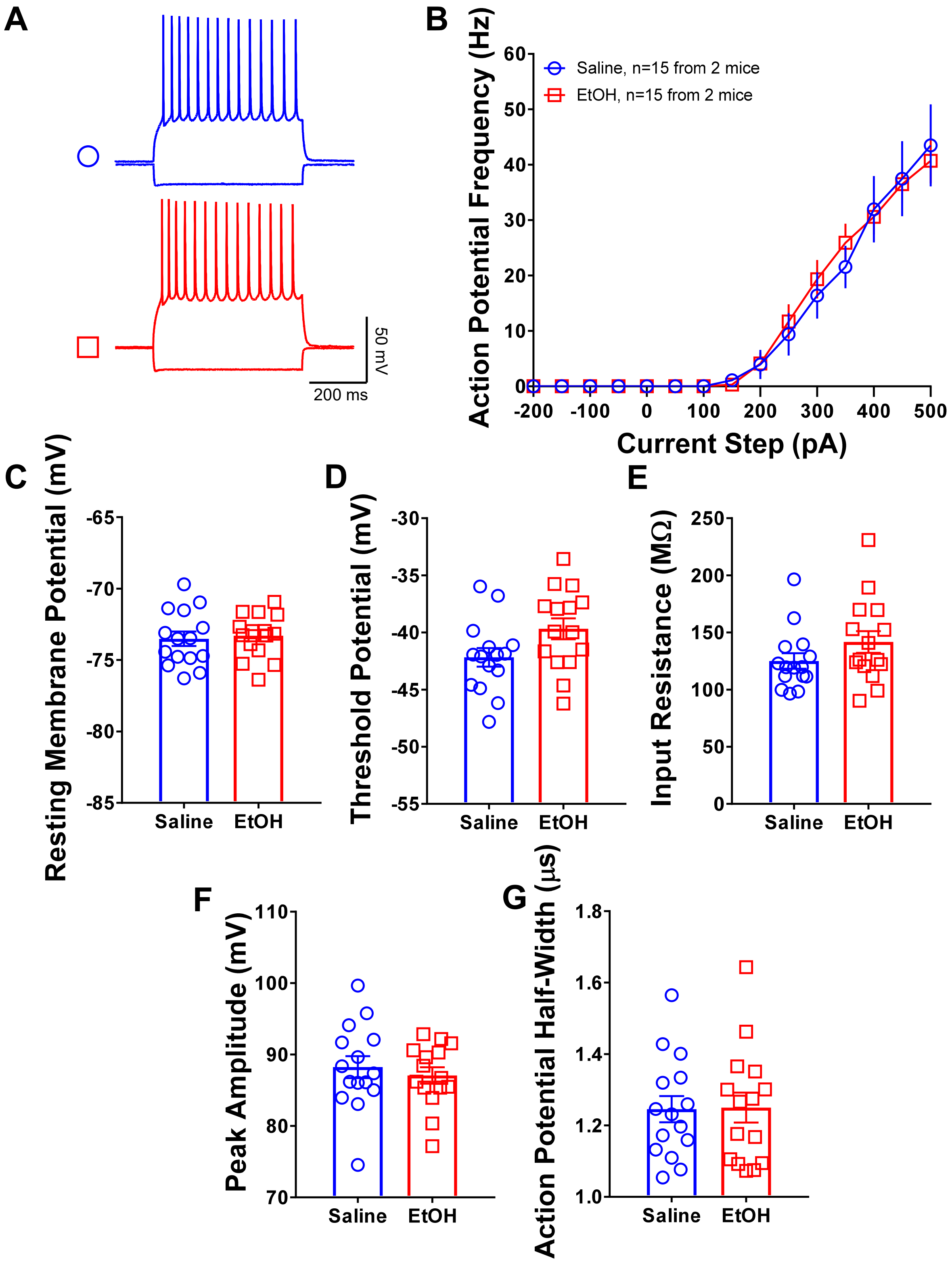
A
50 mV
200 ms
B
Saline, n=15 from 2 mice
EtOH, n=15 from 2 mice
Action Potential Frequency (Hz)
Current Step (pA)
C
Resting Membrane Potential (mV)
D
Threshold Potential (mV)
E
Input Resistance (MΩ)
F
Peak Amplitude (mV)
G
Action Potential Half-Width (µs)
Saline
EtOH

Supplementary Figure 1. A single *in vivo* exposure to ethanol does not affect baseline excitability properties. C57BL/6J mice were injected (intraperitoneal) with saline or EtOH (2 g/kg). 24 h after this injection, intrinsic excitability in MSNs from the DMS were recorded. **A)** Representative action potential traces from MSNs stimulated with 200 pA of current clamp from saline- (blue traces) and EtOH- (red traces) injected mice. **B- G)** No changes in the excitability properties of DMS MSNs after EtOH exposure (n=15 neurons from 2 mice). Data represent mean ± SEM.
